# Telomerase and drug resistance in cancer

**DOI:** 10.1007/s00018-017-2573-2

**Published:** 2017-06-16

**Authors:** Natalia Lipinska, Aleksandra Romaniuk, Anna Paszel-Jaworska, Ewa Toton, Przemyslaw Kopczynski, Blazej Rubis

**Affiliations:** 1 Department of Clinical Chemistry and Molecular Diagnostics, Poznan University of Medical Sciences, Poznan, Poland; 2 Centre for Orthodontic Mini-implants at the Department and Clinic of Maxillofacial Orthopedics and Orthodontics, Poznan University of Medical Sciences, Poznan, Poland

**Keywords:** Telomerase, Telomeres, Cancer, Drug resistance, G-quadruplex stabilizers, Telomere shortening, hTERT expression

## Abstract

It is well known that a decreased expression or inhibited activity of telomerase in cancer cells is accompanied by an increased sensitivity to some drugs (e.g., doxorubicin, cisplatin, or 5-fluorouracil). However, the mechanism of the resistance resulting from telomerase alteration remains elusive. There are theories claiming that it might be associated with telomere shortening, genome instability, hTERT translocation, mitochondria functioning modulation, or even alterations in ABC family gene expression. However, association of those mechanisms, i.e., drug resistance and telomerase alterations, is not fully understood yet. We review the current theories on the aspect of the role of telomerase in cancer cells resistance to therapy. We believe that revealing/unravelling this correlation might significantly contribute to an increased efficiency of cancer cells elimination, especially the most difficult ones, i.e., drug resistant.

## Introduction

Lack of drug sensitivity of cancer cells and their ability to acquire resistance to anti-cancer drugs is one of the most challenging problems in modern oncology. Theoretically, we know the basis of this process (at least one of the mechanisms), which is mainly associated with a cross type of drug resistance that is driven by ABC family (ATP-binding cassette) members, e.g., PgP, BCRP, LRP, MRP, and ca 40 others [1]. It was also reported that an increased DNA repair in cancer cells (mismatch repair pathway activation after the damage provoked by a drug) is another defense mechanism in those cells [2].

The mechanism of drug resistance differs for various cancer types and depends on the category of a drug used in therapy. However, most anti-cancer agents contribute to the generation of reactive oxygen species (ROS) followed by target cells apoptosis. Nevertheless, continuous treatment with the same drug may result in less efficient ROS production that may lead to drug resistance. Importantly, even drugs that directly bind DNA and induce its damage usually require an increased ROS signaling that may be necessary for excessive DNA damage and apoptosis.

Latest reports reveal that drug resistance may also result from an increased translocation of telomerase hTERT (telomerase reverse transcriptase) subunit to mitochondria or translocation of another telomerase-associated factor (telomerase-associated protein 1, TEP1) to vaults. As reported, mitochondrial enrichment with hTERT may be accompanied by ROS level modulation or an increased number of copies of mtDNA that may be followed by apoptosis repression and may play a protective role during therapy [3, 4]. We also know that one of the characteristic features of cancer cells is an increased telomerase expression or activity (circa 95% of cancers, including cancer stem cells) [5, 6].

Numerous studies indicate a relation between telomerase and sensitivity of cancer cells to therapy. This observation is confirmed by the fact that an increased expression of telomerase in cancer cells correlates with their resistance to drugs [7]. Simultaneously, inhibition of telomerase activity results in an increased sensitivity of cancer cells (e.g., breast cancer) to doxorubicin (DOX) [8]. Thus, the correlation between drug resistance and telomerase seems to be evident especially since it is observed during the studies of sensitization process (via telomerase expression downregulation) in cancer cells exposed to radiomimetic compounds (i.e., DNA damaging) [9]. This aspect is highly interesting, because telomerase is responsible for genome stabilization. Apparently, after therapy accompanied by telomerase blocking, we might observe a synergistic process based on two mechanisms—DNA damage (drugs) and mismatch repair/genome stabilization attenuation (telomerase inhibition). Consequently, it might lead to an increased efficacy in cancer cell elimination. Surprisingly, after chemotherapy, the activity of telomerase was reported to increase in LoVo (colon cancer) cells that were accompanied by an increase in telomere length and induction of telomerase subunit expression hTERT and hTR (human telomerase RNA or TERC) [10]. This might implicate a protective role of telomerase in cancer cells exposed to drugs. The efficacy of telomerase and telomeres targeting in cancer therapy is perceived as a promising strategy and is being studied in a broad range of approaches (Table 1).

**Table 1:** Overview of the key telomerase therapeutic strategies directed against telomerase and telomeres [30], modified

Strategy	Factor	Target	Mechanism	References
ASO	GRN163L	hTR	Expression inhibition	[11]
RT inhibition	AZT azidothymidine	DNA elongation	Replication termination	[12]
	BIBR 1532	RT	Non-competitive inhibition	[13]
Immunotherapy	GV1001	HLA	CD4+ stimulation	[14]
	Vx-001	HLA-A		[15]
	GRNVAC1/2 (AST-VAC1/2)	Dendritic cells	Antigen presentation	[16, 17]
G-quadruplex stabilization	BRACO-1910	Telomeric DNA	Access blocking	[13]
	RHPS4			[18]
	TMPyP4			[19]
	Telomestatin			[20]
	Quarfloxin/CX-3543^a^	Telomeric DNA	rRNA biogenesis inhibition^a^	[21]
Uncapping mimicking	T-oligo	Telomeric overhang	Apoptosis, and autophagy induction	[22]
RNAi	hTERT-siRNA hTERC-siRNA	hTERT hTERC	Expression repression	[23, 24]
Preventing binding of telomerase to telomere	IWR1, IWR2, JW55, flavone, XAV939	Tankyrase inhibition	TRF1 dissociation from telomeres	[25]
Nitroreductase-based gene therapy	CB1954	DNA crosslinks	Suicide gene therapy	[26]
Carboxypeptidase-based gene therapy	ZD2767P	DNA-damaging alkylation		[27]
Oncolytic viruses	Telomelysin (OBP-301)	hTERT positive cells	Cancer cell lysis	[28]
Gene therapy	CRISPR/CAS9	hTERT hTERC	Expression repression	[29]

All strategies aim to stop the main telomerase subunit expression, complex activity, or prevent substrate access to provoke cancer cell senescence, death, or proliferation of target cells

*ASO* antisense oligonucleotides, *RT* reverse transcriptase

^a^Requires verification in further studies

However, some questions still appear, e.g., if the efficacy is an effect specific for the inhibitor or for the way of telomerase inhibition/downregulation? Is this effect similar when the key telomerase subunit hTERT is downregulated at the level of expression? Or is the same effect observed when telomeres are shortening? How are signaling pathways modulated at the same time (also in the context of genome destabilization)? How are those pathways modulated when drug therapy is accompanied by telomerase downregulation?

The association of telomerase and mismatch repair pathway seems to be related if we consider the fact that MSH2 (protein element of the DNA mismatch repair complex) binds hTERT promoter leading to its activation, and most probably to an increased expression of the key telomerase subunit hTERT, that is especially important during carcinogenesis [31]. Surprisingly, studies concerning telomerase inhibition with BIBR1532 show no cumulative effect of telomerase inhibition and DOX in drug-resistant cells [32]. Thus, a question arises, what is the mechanism of the correlation between drug sensitivity, drug resistance, and telomerase expression/activity, but also with the resulting mechanism of DNA damage leading to cancer cell death.

## The role of hTERT translocation in cancer resistance to drugs

The hTERT gene encodes telomerase reverse transcriptase, the catalytic subunit of telomerase. As demonstrated, this is one of the two key telomerase subunits that enable enzyme activity, detected mainly in cancer or stem cells [33]. The main function of hTERT is a reverse transcriptase activity that adds a six-base DNA repeat onto chromosome ends and prevents their shortening during successive cell divisions. Telomerase is associated with cell immortality and cancer, which may be related to the ability of hTERT to prevent apoptosis by stabilizing telomeres. However, fundamental information concerning the antiapoptotic function of hTERT is lacking, including a crucial question—whether its activity and/or nuclear localization are required and where telomerase acts to suppress the cell death process. It was demonstrated that overexpression of wild-type human TERT in HeLa cells, and in cells lacking hTERT but containing the telomerase RNA template, increases their resistance to apoptosis induced by the DNA damaging agent etoposide or the bacterial alkaloid staurosporine [34]. In contrast, hTERT mutants with disruptions of either the RT domain or a 14-3-3 binding domain fail to protect cells against apoptosis. Similarly, overexpression of hTERT in cells lacking the telomerase RNA template was also ineffective in preventing apoptosis. Another finding shows that hTERT suppresses apoptosis at an early step before the release of cytochrome c and apoptosis-inducing factor from mitochondria, suggesting that hTERT can suppress a nuclear signal(s) that is an essential component of apoptotic cascades triggered by diverse stimuli.

## hTERT in response to stress

Mitochondria are the major source of ROS, which are mainly produced through the respiratory electron transport chain. Normally, intracellular ROS are dynamically balanced. When cells are exposed to oxidative stress, the endogenous production of ROS exceeds the capacity of the cellular antioxidant defenses, resulting in chemical damage of mtDNA. Mitochondrial DNA contains 37 genes encoding 13 structural proteins that are subunits of various respiratory chain complexes, 22 tRNAs, and two rRNAs. ND1 and COXII, which are encoded by mtDNA, are important components of respiratory chain complexes I and IV. Mitochondrial TERT has been shown to act as a TERC-independent reverse transcriptase and to exhibit RNA-dependent DNA polymerase activity using mitochondrial tRNA as a template [35]. Mitochondrial TERT can bind to the RNA module of mitochondrial RNA processing endoribonuclease (RMRP) and form a complex similar to RNA-dependent RNA polymerase, which affects gene silencing at the post-transcriptional level [36, 37]. Other results suggest that mitochondrial TERT is involved in the regulation of COXII, a subunit of respiratory chain complexes [38].

**Fig. 1:**
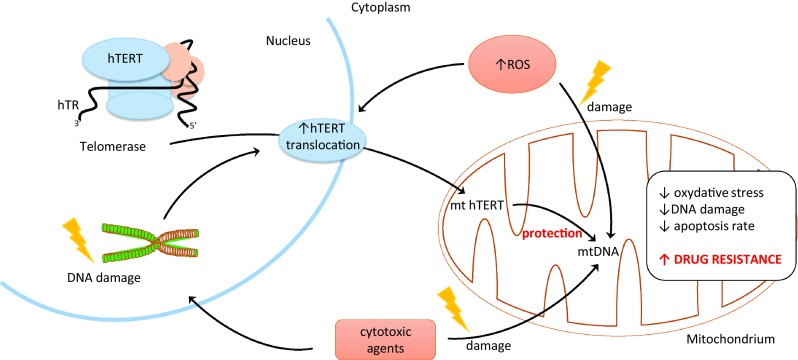
Role of hTERT translocation in cancer

For the first time, the association between telomerase key subunit and cell resistance to stress was shown in 2003. More precisely, it was an oxidative stress. Authors revealed that not only telomerase activity but specifically the C-terminus of hTERT plays a role in hTERT-mediated cellular resistance to oxidative stress. As demonstrated, a 27-kDa hTERT C-terminal polypeptide (hTERTC27) devoid of domains required for telomerase activity was capable of nuclear translocation/telomere-end targeting. It was reported that a low level of hTERTC27 renders hTERT positive HeLa cells sensitive to H2O2-induced oxidative stress and subsequent cell senescence [upregulation of the cyclin/cdk inhibitor p21 (Waf1)]. Most importantly, no alteration in the expression of endogenous hTERT, significant telomere shortening, or inhibition of telomerase activity was observed [39]. Another studies indicated the role of mitochondrial translocation of hTERT in resistance to drugs of human hepatocellular carcinoma (HCC) cells. As demonstrated, using three HCC cell lines (with different resistance index to cisplatin), the apoptosis rates in drug-resistant cells were significantly reduced. Cell-cycle analysis revealed the ratio of drug-resistant cells in *G*2/*M* and S phases increased, while that in *G*1 phase decreased. This process was accompanied by shortening of telomere length in drug-resistant cells under the chemotherapeutic stress. A reduction of damaged mtDNA with the increase in resistance index was revealed indicating protective effect of the mitochondrial translocation of hTERT. Therefore, it may suggest that the mitochondrial translocation of hTERT increases in multidrug-resistant cells and exerts protective effect on mitochondrial function which suggests that drug-resistant tumor cells escape from apoptosis through hTERT-mediated mitochondrial protection. Thus, it might be concluded that mitochondrial translocation of hTERT may serve as an underlying mechanism of MDR (Fig. 1) [40].

As reported elsewhere, mitochondrial translocation of hTERT led to chemotherapeutic resistance in HEPG2 cells. As suggested, phenomenon might result from the contribution of hTERT to reducing ROS production as well as mtDNA damage [18]. In the studies performed in human leukemia cell line K-562, it was shown that telomerase activity inhibition by curcumin (measured using the TRAP assay) is due to suppression of translocation of hTERT subunit, from cytosol to nucleus. More importantly, this process was accompanied by induction of apoptosis in studied cells [41]. The molecular mechanisms by which telomerase demonstrated a pro-survival function resulting in an increased resistance against DNA damage and decreased apoptosis induction remains elusive. It is still unclear whether it is associated with telomere maintenance or is rather a non-telomeric function of the telomerase protein, hTERT. However, the fact is that the protein subunit of telomerase can shuttle from the nucleus to the mitochondria upon oxidative stress. Consequently, it protects mitochondrial function and decreases intracellular oxidative stress. In addition, a significant correlation between nuclear localization of telomerase and high DNA damage was found. At the same time, cells which excluded telomerase from the nucleus displayed no or very low DNA damage. It is known that nuclear DNA damage can be caused by mitochondrially generated reactive oxygen species (ROS). It was shown that the mitochondrial localization of telomerase specifically prevents nuclear DNA damage by decreasing levels of mitochondrial ROS. This decrease of oxidative stress might be a possible cause for high stress resistance of cancer cells and could be especially important for cancer stem cells [42].

## The role of vaults in drug resistance and association with telomerase

It has been some time already, since vaults were reported to contribute to the resistance of cancer cells to drugs. The vault complex, a large-sized ribonucleoprotein, was first described by Kedersha and Rome [43]. The barrel-shaped structures were identified in preparations of clathrin-coated vesicles from rat liver. Their name comes from the fact that they resemble a morphology similar in shape to the vaulted ceilings in cathedrals. Such structures with similar dimension, morphology, and composition are found in cells of diverse eukaryotic organisms including mammals [44, 45]. Since they reveal a high degree of evolutionary conservation, it is suggested that they play an important cellular function.

Vaults’ components are almost ubiquitously expressed and have been implicated in the regulation of several cellular processes including intracellular transport, signal transduction, and immune response. However, most importantly, they also contribute to chemotherapy resistance [46]. Consequently, they are considered to play a protective role against xenobiotics and other stressing factors. The mammalian vault complex consists of multiple copies of three proteins: *M*_r_ 100,000 (MVP), 193,000 (VPARP), and 240,000 (TEP1) (*M*_r_, relative molecular mass). It also contains small untranslated RNA molecules of 88–141 bases.

## Components of the vault complex

### MVP

The major vault protein (MVP, 110 kDa) was found to be identical to the previously described lung resistance-related protein (LRP) [47] and constitutes over 70% of the total mass of the complex. One of the mechanisms leading to drug resistance is based on the ability to pump drugs away from intracellular drug targets through exocytotic vesicles or pump molecules. In addition, MVP/LRP is one of those pumps. The LRP gene is located on chromosome 16, close to the genes coding for the multidrug resistance-associated protein, MRP1. Overexpression of LRP constitutes a prediction of a poor response to chemotherapy in acute myeloid leukemia and ovarian carcinoma. As demonstrated, human MVP promoter lacks a TATA box, as well as other core promoter elements, but harbors putative transcriptional factor binding sites for Sp1 but also p53, STAT1, MyoD, GATA, and YB-1. Consequently, diverse transcription factors are involved in the regulation of MVP in different cell types. Some reports show that MVP expression is induced by a variety of cellular stresses such as DNA damaging agents, UV irradiation, hypoxia, and hyperthermia. Consequently, its role in radiotherapy response was also postulated [48]. Kowalski et al. [49] reported that MVP was involved in host resistance to infection with *Pseudomonas aeruginosa*. Ryu et al. [50] demonstrated that MVP enhanced the resistance of cells to apoptosis induced by H2O2 in senescent human diploid fibroblasts. These findings suggest that MVP plays an important role in cellular responses to stress.

MVP protein and mRNA levels were found to be upregulated in SW-620 cells after treatment with doxorubicin (DOX), etoposide (VP-16), cis-platinum (II) diammine dichloride (CDDP), or SN-38 at their IC50 concentration. Moreover, DOX could directly induce the transcriptional activity of MVP promoter regions, but it did not affect the stability of MVP mRNA. Interestingly, analysis of *MVP* knockout animal derived tissues indicated that the absence of MVP resulted in dramatically lowered cellular levels of other vaults’ component, VPARP [51].

### VPARP

The vault poly-(ADP-ribose) polymerase (VPARP, 193 kDa) contains a functional poly-(ADP-ribose) polymerase (PARP) domain [52]. VPARP is capable of ADP-ribosylating itself and the MVP, but this activity was not yet shown to be of functional importance within the vault complex.

VPARP was originally identified as a minor protein component of the vault ribonucleoprotein particle, which may be involved in molecular assembly or subcellular transport. In addition to the association of VPARP with the cytoplasmic vault particle, subpopulations of VPARP localize to the nucleus and the mitotic spindle, indicating that VPARP may have other cellular functions. VPARP was also found to be associated with telomerase activity and interacted with exogenously expressed telomerase-associated protein 1 (TEP1) in human cells. Experiments with mice deficient in mVparp showed that the animals were viable and fertile for up to five generations, with no apparent changes in telomerase activity or telomere length. Vaults purified from mVparp-deficient mouse liver appeared intact, and no defect in association with other vault components was observed. Mice deficient in mTep1, whose disruption alone does not affect telomere function but does affect the stability of vault RNA, showed no additional telomerase or telomere-related phenotypes when the mTep1 deficiency was combined with an mVparp deficiency. These data suggest that murine mTep1 and mVparp, alone or in combination, are not necessary for normal development, telomerase catalysis, telomere-length maintenance, and vault structure in vivo [53]. There are no data concerning human cells deprived VPARP, and noteworthy, telomeres and telomerase metabolism in those two species differ substantially.

### TEP1

Telomerase-associated protein (TEP1, 240 kDa) was primarily found to be associated with the telomerase complex [54], where its function remains still unknown. TEP1 is an RNA binding protein that is not restricted to the telomerase complex and plays a redundant role in the assembly or localization of the telomerase RNP in vivo [55]. It was also shown to interact specifically with the vault RNAs (vRNAs) [52]. Since only two components of the telomerase complex seem essential for its function in vitro, i.e., hTERT and TR, TEP1 was thought to be a structural component [56, 57]. Indeed, analysis of a TEP1-deficient mouse model showed that this protein is not essential for telomerase activity. Moreover, telomere length was also unaffected after disruption of *TEP1* [55]. However, contribution of TEP1 to telomere elongation in human is still postulated [58]. Although not much is known about the function of TEP1, it was shown that polymorphisms of that gene may correlate with some types of cancer risk, e.g., bladder cancer [59].

## Vaults: summary

It seems that vaults are new players in the field of drug resistance of cancer cells. They are not new, but it looks that novel functions have been assigned to them recently. The MVP components were shown to be associated with the insulin-like growth factor-1, hypoxia-inducible factor-1 alpha, and DNA double-strand break repair systems including non-homologous end joining and homologous recombination. Furthermore, MVP has been proposed as a useful prognostic factor associated with radiotherapy resistance [48].

Vaults are abundantly present in the cytoplasm of eukaryotic cells and they were found to be associated with cytoskeletal elements as well as occasionally with the nuclear envelope. Vaults and MVP have been connected with several cellular processes which are also involved in cancer development like cell motility and differentiation. Due to the overexpression of MVP in several *P*-glycoprotein-negative chemoresistant cancer cell lines (*P*-gp/neg), vaults have been linked to multidrug resistance (MDR). Accordingly, high levels of MVP were found in tissues chronically exposed to xenobiotics. In addition, the expression of MVP correlated with the degree of malignancy in certain cancer types, suggesting a direct involvement in tumor development and/or progression. Based on the finding that MVP binds several phosphatases and kinases including PTEN, SHP-2 as well as Erk, a hypothesis arises that MVP might be involved in the regulation of important cell signaling pathways including the PI3K/Akt and the MAPK pathways [60]. It is not obvious that telomerase or any subunit of the complex plays a pivotal role in those processes, but TEP1 is for sure a common component of both, vaults and telomerase.

## Telomerase and ABC-related drug resistance

Numerous reports show that in most cancer cases that do not respond to therapy, an abundant expression of ABCB1, ABCC1, or ABCG2 appears as poor prognostic factor [32]. These proteins function as membrane transporters thus protect cells from potentially toxic substances and participate in the distribution of drugs. Changes in the expression and activity of ABC proteins may lead to an increased pumping out of drugs from cells and hence a reduction of the effective concentration of the drug in cancer cell. ABC transporters are often found to be inherently expressed in a wide variety of stem cells, where they provide improved protection from toxins [61].

Cells gain the ability to acquire drug resistance at early stage of tumorigenesis process. Yague et al. [62] have shown that it is possible to obtain drug-resistant cells by altering only expression of telomerase, p53, and pRb. The state of the art does not confirm if there is a direct correlation between telomerase expression and ABC-related drug resistance. However, in the literature, there are some examples of attempts to associate telomerase with drug resistance related to ABC overexpression. The concept of such association is based on work of Wang et al. [63], who showed a prognostic value and correlation of telomerase expression with expression of genes associated with multidrug resistance in lung cancer patients. On the other hand, Sakin et al. [64] suggested a lack of such correlation in an in vitro study performed in breast cancer cells. A tenuous correlation was shown in melanoma cells revealing co-expression of ABC transporters, ABCB5 and ABCC2 and hTERT [65]. Smith et al. [66] assessed a panel of human tumor cell lines resistant to vindesine, gemcitabine, and cisplatin, and disclosed that all have displayed changes in telomerase activity and/or telomere length compared to their parental lines. Recent findings suggest that signal transducer and activator of transcription 5 (STAT5) connects these two mechanisms through binding to the promoter regions of both the human TERT gene and the MDR1 gene [67, 68].

Another study shows that doxorubicin treatment of imatinib resistant cell line with *P*-glycoprotein overexpression was accompanied by an increased phosphorylation of BCR-ABL and STAT5, as well as increased hTERT expression. Interestingly, silencing of STAT5 expression reduced both the expression of *P*-glycoprotein and telomerase activity and restore sensitivity to imatinib in drug-resistant CML cells [67, 68]. A direct regulation of the MDR pathways and telomerase by interpheron-alpha (IFN-alpha) (used in the therapy of advanced cutaneous melanoma) has also been hypothesized. Treatment of melanoma cells with IFN-alpha, the MDR genes regulator, revealed no correlation between hTERT and TEP1 mRNA expression, whereas significant positive correlations were found between TEP1 and MDR1 mRNA, and between TEP1 and LRP/MVP mRNA [69]. Alternative results were reported by Gomez et al. [70]. They showed that in A549 derived clones resistant to a DNA-alkylating agent (ethyl methanesulfonate), and selected for resistance to 12549 (telomerase inhibitor; a potent G-quadruplex ligand), hTERT expression was significantly increased (accompanied by an increased telomerase activity) but with no multidrug resistance phenotype alterations. As reported, no variations in multidrug-related protein 1 (MRP1) and breast cancer resistance protein (BCRP) transcripts were observed while multidrug resistance 1 (MDR1) transcript was undetectable. It was also suggested that resistance to 12549 (or other G-4 ligands, i.e., telomestatin or BRACO-19) was associated with both, upregulation of telomerase expression/activity and alteration of telomere capping functions that may participate directly or indirectly in the mechanism of resistance in some cell types. Thus, a direct correlation of telomerase modulation and resistant phenotype acquisition was revealed but in a non-ABC-related mechanism.

## Resistance mechanism specific to G-quadruplex inhibitors

There is area of telomerase/telomere-based cancer strategy that focuses on the use of G-quadruplex stabilizers that prevent telomerase access to its substrate, and it constitutes a promising expansion. Eventually, it appears that G-quadruplex stabilizers play some more complex functions. Gomez et al. reported a potential correlation of telomerase and cancer cells resistance to G-quadruplex ligands that might be associated with a subtle balance between telomerase inhibition and telomere uncapping. These studies provided an evidence that telomerase activity and telomere length are key cellular determinants of the resistance phenotype [70, 71]. Moreover, cancer cells resistance to G-quadruplex ligands seem to be selective to this group of compounds that was confirmed by a cross resistance (as mentioned above) with other telomerase inhibitors with no correlation to other anti-cancer agents showing alternative mechanisms of action. An interesting report shows that ROS activation of PPM1D/WIP1 (DNA damage signaling pathway responsible for phosphorylation status of Chk1 and γ-H2AX) by 12459 in A549 cells may lead to an inhibition of DNA repair signaling that triggers telomeric dysfunction or other genomic DNA damages but that finally leads to a delayed cell death [72]. In addition, the relation between the way of treatment and observed effect (including the initiated mechanism) was observed implicating an effect dependent on variability of factors including long-/short-term treatment, concentration as well as the chemical structure of the ligand or the nature of the studied cell line. Noteworthy, the biological effect of G-quadruplex stabilization that is associated with a specific degradation of the telomeric overhang seems to be independent of the Bcl-2 expression status. Altogether, it gives an impression that telomere/telomerase targeting drugs trigger effects that are probably more directly associated with DNA stability, damage, and repair pathways but also with the mechanism of action of the therapeutic compound, e.g., direct DNA targeting or stress provoking (including oxidative stress) [73]. It is clear that although telomere length in humans is determined by genetic predispositions, it is also significantly affected by environmental factors that altogether results in different (individual) attrition rate. It is also evident that direct modulation of telomerase activity by inhibitors, quadruplex stabilizers, repressors or ROS, and other stress factors (including psychological and life stress) affect telomere length. Consequently, even smoking (an excellent example for higher ROS formation) leads to a progressive shortening of telomeres. Especially, the GGG triplet within the human telomere sequence (TTAGGG) is particularly vulnerable to chemical modifications. Thus, telomere or telomerase targeting may ultimately lead to genomic instability. Some authors claim that on one hand, it may be used in cancer therapy, but on the other hand, it may contribute to initiation of carcinogenesis [74].

## Telomerase-positive and ABC genes expressing cancer stem cells (CSC): neoplasm maintenance

Years of research work in the field of cancer biology confirmed highly heterogenic character of cancer cell populations [75]. A variety of types of cells differing in morphology as well as in molecular status were observed within malignant tumor or among the leukemic cells in circulation of one patient. It is believed that most, if not all, tumors contain a small sub-population of cancer stem cells (CSC), called also cancer stem-like cells or tumor-initiating cells [76]. CSCs share some features with normal adult stem cells. Both types of cells exhibit high levels of telomerase activity, increased level and/or activity of transmembrane ATP-binding cassette (ABC) transporter proteins, and possess the ability to self-renewal and differentiation, induction of antiapoptotic mechanisms, and migration potential [77, 78]. Cancer stem cells are able to reproduce themselves and their presence in the heterogenous neoplastic cells population enables sustaining the cancer. CSCs can “differentiate” into nontumorigenic mature cancer that drives the tumor growth and spread [79].

One of the theories explaining the immortality of tumor cells proposes that cancer originates from normal stem cells, which show constitutive telomerase activity. According to this cancer stem cell model of carcinogenesis, the event that initiates neoplastic transformation occurs in normal stem cell [80–82]. There are scientific evidences proving that during aging, stem cells can accumulate so many mutations that the scenario where nontumorigenic normal stem cell becomes tumor-initiating cell is very likely [83]. Undoubtedly, telomerase is crucial for cancer stem cell existence. Studies with the use of animal experimental model (Sca1-BCRABLp210) with the established telomerase deficiency revealed that telomerase presence is necessary for the generation and maintenance of cancer stem cells. Complete deletion of telomerase coding gene or even loss of one allele of the gene resulted in prevention of BCR-ABL-induced chronic myeloid leukemia (CML) development [82]. Interestingly, one of the key antiapoptotic proteins—Bcl-2—is not required to sustain leukemic stem cells population in mice [84]. Inhibition of telomerase activity with the use of Imetelstat caused a significant reduction of clonogenic growth of CD138^−^ cancer stem cells isolated from patients with myeloma [85]. There are reports showing that some cancer stem cells and mature cancer cells have short telomeres while exhibiting significant telomerase activity [86]. These observations may indicate that telomerase in cancer stem cells may play some other function(s) than only telomere lengthening.

Another important feature of cancer stem cells is their intrinsic drug resistance related to overexpression of genes encoding ABC transporter proteins. It is a serious problem from the clinical and pharmacological point of view, because CSCs, that are rather rare in the tumor mass, survive the therapy (chemo- and radiotherapy) and propagate giving rise to new tumor. Cancer stem cells are characterized by high resistance to antimitotic drugs resulting from the upregulation of drug efflux pumps. ABCB1, ABCG2, and ABCB5 transmembrane proteins are listed among CSC’s markers [87]. In human melanoma cells, coexistence of ABCB1, ABCG2, and ABCC2 together with stem cell markers on the cell surface was observed in a subset of cells. Moreover, it was reported that in melanoma stem cells, concomitant occurrence of ABCG2 and CD133 can be a stemness marker [88]. CSCs isolated from pancreatic cancer cell line PANC1 were verified as cells expressing both *ABCB1* and *ABCG2* [89]. In addition, the ability of CSCs to evade chemo- and radiotherapy is enhanced by the fact that the cells are relatively quiescent and slow cycling. These two features protect them against drugs targeting rapidly proliferating cells [87].

Concluding, telomerase-positive cancer cells with stem-like properties are considered the most resistant subset of cells within the malignant tumor. One of the extratelomeric function of telomerase is an interplay with molecules regulating gene expression. TERT (telomerase subunit) is hypothesized to act as a transcription factor and play a key role in the modulation of many processes related to neoplastic transformation, induction of cancer stemness as well contribution to the drug resistance development [90].

## Sensitization of cancer cells through telomerase inhibition

Numerous studies reported a correlation between telomerase inhibition and sensitization of cancer cells. As demonstrated, pharmacological inhibition of telomerase catalytic activity by BIBR1532 sensitized both drug-sensitive and drug-resistant leukemia and breast cancer cells; moreover, these effects were telomere-length-dependent [32]. Another telomerase inhibitor, imetelstat, sensitized primary CLL lymphocytes to fludarabine in vitro [91]. Treatment of flavopiridol-resistant cells with G-quadruplex stabilizer BRACO-19 alone also led to a rapid inhibition of cell growth that was not observed in the parental cell line. It is noteworthy that combination of BRACO-19 and flavopiridol caused a threefold reduction in cell proliferation compared to the use of flavopiridol alone. Interestingly, this effect was not observed in parental colon cancer cell line that was sensitive to the flavopiridol and showed lower telomerase activity [92]. Treatment with doxorubicin and taxol combined with inhibition of telomerase increased senescence and apoptosis of breast cancer cells. Another study revealed that telomerase inhibition itself, not necessarily short telomeres, was crucial for sensitization in treated cells [9]. Similarly, telomere-length independent cellular response was observed by Park et al. [93]. Knockdown of hTERT in resistant bladder cancer cell line increased cisplatin-induced cell death and accelerated the translocation of Bax into the mitochondrial membrane and the release of cytochrome C. Thus, once more, telomerase was suggested to play an important role in mitochondrial metabolism during the process of intrinsic apoptosis.

## Telomerase and DNA damage/apoptosis resistance

Multiple studies show that telomeres and telomerase are centrally involved in the capability for an unlimited proliferation of cells [94]. Since telomeres are essential for maintaining and protecting chromosomes from degradation, telomere dysfunction is considered to play a crucial role in initiating genomic instability. Abnormalities in telomere function lead to chromosome rearrangements, breakage–fusion–bridge cycles (BFB), and the generation of novel chromosomal variants. As revealed, in the presence of intact p53, critically short telomeres induce cell-cycle arrest, cellular senescence, or apoptosis what suppress tumorigenesis. Dysfunctional telomeres are also recognized as double-strand DNA breaks, activating the DNA damage response (DDR) checkpoints, including ataxia telangiectasia mutated (ATM) and checkpoint kinase 2 (CHK2) [95]. Thereby, telomere shortening decreases lifespan and functions as a barrier to cancer development.

As suggested, telomerase itself has a pro-survival function resulting in an increased resistance against DNA damage and decreased apoptosis induction [2]. Rubio et al. [96] showed that telomerase confers resistance to ionizing radiation, bleomycin, hydrogen peroxide, and etoposide in human fibroblast cells with short, near-dysfunctional, telomeres. This resistance depends on the ability of telomerase to elongate the short telomeres. Moreover, the authors revealed that telomerase did not reduce genotoxic stress in cells with long telomeres. As mentioned already, telomerase exclusion from nucleus into mitochondria could be one of the possible non-telomeric mechanisms that decrease nuclear DNA damage and apoptosis caused by an anti-cancer treatment. Beside the decrease of mitochondrial ROS level, telomerase can also disrupt DNA repair processes through interference with DNA repair enzymes under DNA damage conditions [42]. This might contribute to increased resistance of cancer cells to various anti-cancer treatments.

Several reports show that hTERT might play an important role in direct inhibition of at least two cell death pathways. It was shown that hTERT depletion upregulates the induction of apoptotic cell death by cisplatin, etoposide, mitomycin C, and reactive oxygen species in cervical and colon cancer cell lines. Interestingly, inhibition of mitochondrial membrane permeabilization precluded the induction of cell death by the combination of hTERT downregulation and chemotherapeutic agents. These findings indicate that hTERT functions as an endogenous inhibitor of mitochondrial cell death pathway [97].

**Fig. 2:**
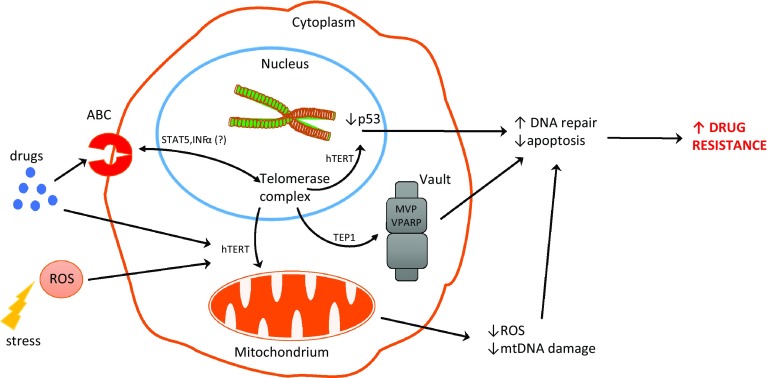
Potential interactions between telomerase and drug resistance in cancer cells

Ectopic expression of hTERT is responsible for suppressing a p53-dependent cell death induced by DNA-damaging agents. The same effect was observed for both normal and mutant hTERT (without telomerase activity) and allowed cells to be resistant to DNA damage and to suppress activation of p53 [98] (see Fig. 2). Similarly, bortezomib inhibition of hTERT expression in leukemic and gastric cancer cells contributed to telomere dysfunction and cellular apoptosis. In addition, hTERT overexpression protected cells against bortezomib-induced DNA damage [99].

One of the telomere-binding proteins, TRF2, regulates both telomere protection and telomere length as well as interacts with several DNA repair proteins. It was found that TRF2 expression increases after DNA double-strand breaks induction by anti-cancer drugs or irradiation in gastric cancer cells. As demonstrated, TRF2 upregulation was more dramatic in drug-resistant cells, whereas its inhibition by RNAi in drug-resistant cells partially reversed its resistance phenotype [100, 101].

## Conclusions

Telomerase regulation studies concern mainly the relationship between telomerase activity inhibition and cancer treatment. In addition, the impact on the cell biology and the non-telomeric functions of telomerase is also investigated. First observations of connection between telomerase and drug resistance in cancer concerned sensitization of resistant cells after telomerase inhibition. Surprisingly, only imatinib mesylate (telomerase inhibitor) is documented to reveal desired effects in treating CML patients. However, lot of potential telomerase inhibitors still is under research and clinical trials. Direct connection between telomerase (or its components) and ABC transporter expression/function seems to occur only in some types of cancer cells. This may be due to common telomerase but limited ABC genes expression regulation.

We assume that non-canonical functions of telomerase might play a more important role and contribute towards the higher resistance of cancer cells. That is supported by a fact that hTERT translocation between the nucleus and mitochondria can be considered as a negative prognostic factor in cancer cells. Finally, telomerase components are involved in death pathways at various levels, and thus, its regulation may be crucial for the development of death-resistance mechanisms (Fig. 2). Unfortunately, some telomerase-based approaches provoke activation of alternative lengthening of telomerase (ALT), which may underlie the development of another resistant mechanism. Anyway, the correlation of telomerase and drug resistance is a fact. The question is how those processes are associated? However, another question appears—how to use this knowledge to overcome cancer cells resistance? How useful those data could be, partly was already presented in some pioneering studies performed in 3D culture systems. Liu et al. [102] revealed that CaSki sphere-forming cells (exhibiting higher telomerase activity) were more resistant to chemotherapeutic drugs than the control CaSki cells. These studies, however, are in progress but without a doubt should be continued.

## References

[1] Eckford PDW, Sharom FJ (2009). ABC efflux pump-based resistance to chemotherapy drugs. Chem Rev.

[2] Masutomi K, Possemato R, Wong JMY, Currier JL, Tothova Z, Manola JB (2005). The telomerase reverse transcriptase regulates chromatin state and DNA damage responses. Proc Natl Acad Sci USA.

[3] Yan J, Zhou Y, Chen D, Li L, Yang X, You Y (2015). Effects of mitochondrial translocation of telomerase on drug resistance in hepatocellular carcinoma cells. J Cancer.

[4] Niu R, Yoshida M, Ling F (2012). Increases in mitochondrial DNA content and 4977-bp deletion upon ATM/Chk2 checkpoint activation in HeLa cells. PLoS One.

[5] Hiyama E, Hiyama K (2004). Telomerase detection in the diagnosis and prognosis of cancer. Cytotechnology.

[6] Holysz H, Lipinska N, Paszel-Jaworska A, Rubis B (2013). Telomerase as a useful target in cancer fighting-the breast cancer case. Tumour Biol.

[7] Shin J-S, Foo T, Hong A, Zhang M, Lum T, Solomon MJ (2012). Telomerase expression as a predictive marker of radiotherapy response in rectal cancer. Pathology.

[8] Dong X, Liu A, Zer C, Feng J, Zhen Z, Yang M (2009). siRNA inhibition of telomerase enhances the anti-cancer effect of doxorubicin in breast cancer cells. BMC Cancer.

[9] Poynter KR, Sachs PC, Bright AT, Breed MS, Nguyen BN, Elmore LW (2009). Genetic inhibition of telomerase results in sensitization and recovery of breast tumor cells. Mol Cancer Ther.

[10] Kuranaga N, Shinomiya N, Mochizuki H (2001). Long-term cultivation of colorectal carcinoma cells with anti-cancer drugs induces drug resistance and telomere elongation: an in vitro study. BMC Cancer.

[11] Röth A, Harley CB, Baerlocher GM (2010). Imetelstat (GRN163L)–telomerase-based cancer therapy. Recent Results Cancer Res.

[12] Johnston JS, Johnson A, Gan Y (2003). Synergy between 3′-azido-3′-deoxythymidine and paclitaxel in human pharynx FaDu cells. Pharm Res.

[13] Mo Y, Gan Y, Song S (2003). Simultaneous targeting of telomeres and telomerase as a cancer therapeutic approach. Cancer Res.

[14] Shaw VE, Naisbitt DJ, Costello E (2010). Current status of GV1001 and other telomerase vaccination strategies in the treatment of cancer. Expert Rev Vaccines.

[15] Vetsika EK, Konsolakis G, Aggouraki D (2012). Immunological responses in cancer patients after vaccination with the therapeutic telomerase-specific vaccine Vx-001. Cancer Immunol Immunother.

[16] Su Z, Dannull J, Yang BK (2005). Telomerase mRNA-transfected dendritic cells stimulate antigen-specific CD8+ and CD4+ T cell responses in patients with metastatic prostate cancer. J Immunol.

[17] Di Persio JF, Collins RH, Blum W (2009). Immune responses in AML patients following vaccination with GRNVAC1, autologous RNA transfected dendritic cells expressing telomerase catalytic subunit hTERT. ASH Annu Meet Abstr.

[18] Phatak P, Cookson JC, Dai F (2007). Telomere uncapping by the G-quadruplex ligand RHPS4 inhibits clonogenic tumour cell growth in vitro and in vivo consistent with a cancer stem cell targeting mechanism. Br J Cancer.

[19] Mikami-Terao Y, Akiyama M, Yuza Y (2008). Antitumor activity of G-quadruplex-interactive agent TMPyP4 in K562 leukemic cells. Cancer Lett.

[20] Waki K, Anno K, Ono T (2010). Establishment of functional telomerase immortalized human hepatocytes and a hepatic stellate cell line for telomere-targeting anticancer drug development. Cancer Sci.

[21] Drygin D, Siddiqui-Jain A, O’Brien S (2009). Anticancer activity of CX-3543: a direct inhibitor of rRNA biogenesis. Cancer Res.

[22] Yaar M, Eller MS, Panova I (2007). Telomeric DNA induces apoptosis and senescence of human breast carcinoma cells. Breast Cancer Res.

[23] Zhong YQ, Xia ZS, Fu YR (2010). Knockdown of hTERT by SiRNA suppresses growth of Capan-2 human pancreatic cancer cell via the inhibition of expressions of Bcl-2 and COX-2. J Digest Dis.

[24] Li S, Rosenberg JE, Donjacour AA (2004). Rapid inhibition of cancer cell growth induced by lentiviral delivery and expression of mutant-template telomerase RNA and anti-telomerase short-interfering RNA. Cancer Res.

[25] Lu H, Lei Z, Lu Z (2013). Silencing tankyrase and telomerase promotes A549 human lung adenocarcinoma cell apoptosis and inhibits proliferation. Oncol Rep.

[26] Plumb JA, Bilsland A, Kakani R (2001). Telomerase-specific suicide gene therapy vectors expressing bacterial nitroreductase sensitize human cancer cells to the pro-drug CB1954. Oncogene.

[27] Schepelmann S, Ogilvie LM, Hedley D (2007). Suicide gene therapy of human colon carcinoma xenografts using an armed oncolytic adenovirus expressing carboxypeptidase G2. Cancer Res.

[28] Xi L, Schmidt JC, Zaug AJ, Ascarrunz DR, Cech TR (2015). A novel two-step genome editing strategy with CRISPR-Cas9 provides new insights into telomerase action and TERT gene expression. Genome Biol.

[29] Nemunaitis J, Tong AW, Nemunaitis M (2010). A phase I study of telomerase-specific replication competent oncolytic adenovirus (telomelysin) for various solid tumors. Mol Ther.

[30] Xu Y, Goldkorn A (2016). Telomere and telomerase therapeutics in cancer. Genes (Basel).

[31] Kang X, Chen W, Kim RH, Kang MK, Park N-H (2009). Regulation of the hTERT promoter activity by MSH2, the hnRNPs K and D, and GRHL2 in human oral squamous cell carcinoma cells. Oncogene.

[32] Ward RJ, Autexier C (2005). Pharmacological telomerase inhibition can sensitize drug-resistant and drug-sensitive cells to chemotherapeutic treatment. Mol Pharmacol.

[33] Saldanha SN, Andrews LG, Tollefsbol TO (2003). Analysis of telomerase activity and detection of its catalytic subunit, hTERT. Anal Biochem.

[34] Zhang P, Chan SL, Fu W, Mendoza M, Mattson MP (2003). TERT suppresses apoptotis at a premitochondrial step by a mechanism requiring reverse transcriptase activity and 14-3-3 protein binding ability. FASEB J.

[35] Sharma NK, Reyes A, Green P, Caron MJ, Bonini MG, Gordon DM (2012). Human telomerase acts as a hTR-independent reverse transcriptase in mitochondria. Nucleic Acids Res.

[36] Maida Y, Yasukawa M, Furuuchi M, Lassmann T, Possemato R, Okamoto N (2009). An RNA-dependent RNA polymerase formed by TERT and the RMRP RNA. Nature.

[37] Maida Y, Masutomi K (2011). RNA-dependent RNA polymerases in RNA silencing. Biol Chem.

[38] Yan J, Zhou Y, Chen D, Li L, Yang X, You Y (2015). Effects of mitochondrial translocation of telomerase on drug resistance in hepatocellular carcinoma cells. J Cancer.

[39] Huang J, Bai YX, Han SW, Ng SS, Jing DD, Wong BC (2003). A human TERT C-terminal polypeptide sensitizes HeLa cells to H2O2-induced senescence without affecting telomerase enzymatic activity. Biochem Biophys Res Commun.

[40] Ling X, Wen L, Zhou Y (2012). Role of mitochondrial translocation of telomerase in hepatocellular carcinoma cells with multidrug resistance. Int J Med Sci.

[41] Chakraborty S, Ghosh U, Bhattacharyya NP, Bhattacharya RK, Roy M (2006). Inhibition of telomerase activity and induction of apoptosis by curcumin in K-562 cells. Mutat Res Mol Mech Mutagen.

[42] Singhapol C, Pal D, Czapiewski R, Porika M, Nelson G, Saretzki GC (2013). Mitochondrial telomerase protects cancer cells from nuclear DNA damage and apoptosis. PLoS One.

[43] Kedersha NL, Rome LH (1986). Isolation and characterization of a novel ribonucleoprotein particle: large structures contain a single species of small RNA. J Cell Biol.

[44] Kedersha NL, Miquel MC, Bittner D, Rome LH (1990). Vaults. II. Ribonucleoprotein structures are highly conserved among higher and lower eukaryotes. J Cell Biol.

[45] Rome L, Kedersha N, Chugani D (1991). Unlocking vaults: organelles in search of a function. Trends Cell Biol.

[46] Berger W, Steiner E, Grusch M, Elbling L, Micksche M (2009). Vaults and the major vault protein: novel roles in signal pathway regulation and immunity. Cell Mol Life Sci.

[47] Scheffer GL, Wijngaard PLJ, Flens MJ, Izquierdo MA, Slovak ML, Pinedo HM (1995). The drug resistance-related protein LRP is the human major vault protein. Nat Med.

[48] Lara PC, Pruschy M, Zimmermann M, Henríquez-Hernández LA (2011). MVP and vaults: a role in the radiation response. Radiat Oncol.

[49] Kowalski MP, Dubouix-Bourandy A, Bajmoczi M, Golan DE, Zaidi T, Coutinho-Sledge YS (2007). Host resistance to lung infection mediated by major vault protein in epithelial cells. Science.

[50] Ryu SJ, An HJ, Oh YS, Choi HR, Ha MK, Park SC (2008). On the role of major vault protein in the resistance of senescent human diploid fibroblasts to apoptosis. Cell Death Differ.

[51] Mossink MH, van Zon A, Fränzel-Luiten E, Schoester M, Kickhoefer VA, Scheffer GL (2002). Disruption of the murine major vault protein (MVP/LRP) gene does not induce hypersensitivity to cytostatics. Cancer Res.

[52] Kickhoefer VA, Siva AC, Kedersha NL, Inman EM, Ruland C, Streuli M (1999). The 193-kD vault protein, VPARP, is a novel poly(ADP-ribose) polymerase. J Cell Biol.

[53] Liu Y, Snow BE, Kickhoefer VA, Erdmann N, Zhou W, Wakeham A (2004). Vault poly(ADP-ribose) polymerase is associated with mammalian telomerase and is dispensable for telomerase function and vault structure in vivo. Mol Cell Biol.

[54] Harrington L, McPhail T, Mar V, Zhou W, Oulton R, Bass MB (1997). A mammalian telomerase-associated protein. Science.

[55] Liu Y, Snow BE, Hande MP, Baerlocher G, Kickhoefer VA, Yeung D (2000). Telomerase-associated protein TEP1 is not essential for telomerase activity or telomere length maintenance in vivo. Mol Cell Biol.

[56] Weinrich SL, Pruzan R, Ma L, Ouellette M, Tesmer VM, Holt SE (1997). Reconstitution of human telomerase with the template RNA component hTR and the catalytic protein subunit hTRT. Nat Genet.

[57] Beattie TL, Zhou W, Robinson MO, Harrington L (1998). Reconstitution of human telomerase activity in vitro. Curr Biol.

[58] Bhattacharyya S, Sandy A, Groden J (2010). Unwinding protein complexes in ALTernative telomere maintenance. J Cell Biochem.

[59] Chang J, Dinney CP, Huang M, Wu X, Gu J, Blackburn E (2012). Genetic variants in telomere-maintenance genes and bladder cancer risk. PLoS One.

[60] Steiner E, Holzmann K, Elbling L, Micksche M, Berger W (2006). Cellular functions of vaults and their involvement in multidrug resistance. Curr Drug Targets.

[61] Kovalev AA, Tsvetaeva DA, Grudinskaja TV (2013). Role of ABC-cassette transporters (MDR1, MRP1, BCRP) in the development of primary and acquired multiple drug resistance in patients with early and metastatic breast cancer. Exp Oncol.

[62] Yagüe E, Arance A, Kubitza L, O’Hare M, Jat P, Ogilvie CM (2007). Ability to acquire drug resistance arises early during the tumorigenesis process. Cancer Res.

[63] Wang J, Liu X, Fang J (1999). Expression and clinical significance of telomerase catalytic subunit gene in lung cancer and its correlations with genes related to drug resistance and apoptosis. Zhonghua Zhong Liu Za Zhi.

[64] Sakin V, Eskiocak U, Kars MD, Iseri OD, Gunduz U (2008). hTERT gene expression levels and telomerase activity in drug resistant MCF-7 cells. Exp Oncol.

[65] Keshet GI, Goldstein I, Itzhaki O, Cesarkas K, Shenhav L, Yakirevitch A (2008). MDR1 expression identifies human melanoma stem cells. Biochem Biophys Res Commun.

[66] Smith V, Dai F, Spitz M, Peters GJ, Fiebig HH, Hussain A (2009). Telomerase activity and telomere length in human tumor cells with acquired resistance to anticancer agents. J Chemother.

[67] Yamada O, Ozaki K, Furukawa T, Machida M, Wang Y-H, Motoji T (2011). Activation of STAT5 confers imatinib resistance on leukemic cells through the transcription of TERT and MDR1. Cell Signal.

[68] Katsumi S, Kawauchi K, Ozaki K, Shimizu S, Kimura T, Motoji T (2013). Analysis of molecular mechanism involved in development of acute myeloid leukemia. Gan To Kagaku Ryoho.

[69] Maellaro E, Pacenti L, Del Bello B, Valentini MA, Mangiavacchi P, De Felice C (2003). Different effects of interferon-alpha on melanoma cell lines: a study on telomerase reverse transcriptase, telomerase activity and apoptosis. Br J Dermatol.

[70] Gomez D, Aouali N, Londoño-Vallejo A, Lacroix L, Mégnin-Chanet F, Lemarteleur T, Douarre C, Shin-ya K, Mailliet P, Trentesaux C, Morjani H, Mergny JL, Riou JF (2003). Resistance to the short term antiproliferative activity of the G-quadruplex ligand 12459 is associated with telomerase overexpression and telomere capping alteration. J Biol Chem.

[71] Gomez D, Aouali N, Renaud A, Douarre C, Shin-Ya K, Tazi J, Martinez S, Trentesaux C, Morjani H, Riou JF (2003). Resistance to senescence induction and telomere shortening by a G-quadruplex ligand inhibitor of telomerase. Cancer Res.

[72] Douarre C, Mergui X, Sidibe A, Gomez D, Alberti P, Mailliet P, Trentesaux C, Riou JF (2013). DNA damage signaling induced by the G-quadruplex ligand 12459 is modulated by PPM1D/WIP1 phosphatase. Nucleic Acids Res.

[73] Cheung-Ong K, Giaever G, Nislow C (2013). DNA-damaging agents in cancer chemotherapy: serendipity and chemical biology. Chem Biol.

[74] Jäger K, Walter M (2016). Therapeutic targeting of telomerase. Genes (Basel).

[75] Pardal R, Clarke MF, Morrison SJ (2003). Applying the principles of stem-cell biology to cancer. Nat Rev Cancer.

[76] Singh A, Settleman J (2010). EMT, cancer stem cells and drug resistance: an emerging axis of evil in the war on cancer. Oncogene.

[77] Fulawka L, Donizy P, Halon A (2014). Cancer stem cells—the current status of an old concept: literature review and clinical approaches. Biol Res.

[78] Wicha MS, Liu S, Dontu G (2006). Cancer stem cells: an old idea—a paradigm shift. Cancer Res.

[79] Magee JA, Piskounova E, Morrison SJ, Veerman AJ, Huismans DR, Munske L (2012). Cancer stem cells: impact, heterogeneity, and uncertainty. Cancer Cell.

[80] Armanios M, Greider CW (2005). Telomerase and cancer stem cells. Cold Spring Harb Symp Quant Biol.

[81] Günes C, Rudolph KL (2013). The role of telomeres in stem cells and cancer. Cell.

[82] Sánchez-García I, Romero-Camarero I, Sánchez-García I (2012). Understanding telomerase in cancer stem cell biology. Cell Cycle.

[83] Kumar M, Lechel A, Güneş Ç (2016). Telomerase: the devil inside. Genes.

[84] González-Herrero I, Vicente-Dueñas C, Orfao A, Flores T, Jiménez R, Cobaleda C (2010). Bcl2 is not required for the development and maintenance of leukemia stem cells in mice. Carcinogenesis.

[85] Matsui W, Wang Q, Vala M, Barber JP, Meeker A, Tressler R (2015). Cancer stem cell targeting in multiple myeloma by GRN163L, a novel and potent telomerase inhibitor. Blood.

[86] Shay JW, Wright WE (2010). Telomeres and telomerase in normal and cancer stem cells. FEBS Lett.

[87] Moitra K (2015). Overcoming multidrug resistance in cancer stem cells. Biomed Res Int.

[88] Welte Y, Adjaye J, Lehrach HR, Regenbrecht CR (2010). Cancer stem cells in solid tumors: elusive or illusive?. Cell Commun Signal.

[89] Zhou J, Wang C-Y, Liu T, Wu B, Zhou F, Xiong J-X (2008). Persistence of side population cells with high drug efflux capacity in pancreatic cancer. World J Gastroenterol.

[90] Teralı K, Yilmazer A (2016). New surprises from an old favourite: the emergence of telomerase as a key player in the regulation of cancer stemness. Biochimie.

[91] Shawi M, Chu TW, Martinez-Marignac V, Yu Y, Gryaznov SM, Johnston JB (2013). Telomerase contributes to fludarabine resistance in primary human leukemic lymphocytes. PLoS One.

[92] Incles CM, Schultes CM, Kelland LR, Neidle S (2003). Acquired cellular resistance to flavopiridol in a human colon carcinoma cell line involves up-regulation of the telomerase catalytic subunit and telomere elongation. Sensitivity of resistant cells to combination treatment with a telomerase inhibitor. Mol Pharmacol.

[93] Park YP, Kim KD, Kang SH, Yoon D-Y, Park JW, Kim JW (2008). Human telomerase reverse transcriptase (hTERT): a target molecule for the treatment of cisplatin-resistant tumors. Korean J Lab Med.

[94] Blasco MA (2005). Telomeres and human disease: ageing, cancer and beyond. Nat Rev Genet.

[95] Artandi SE, DePinho RA (2010). Telomeres and telomerase in cancer. Carcinogenesis.

[96] Rubio MA, Davalos AR, Campisi J (2004). Telomere length mediates the effects of telomerase on the cellular response to genotoxic stress. Exp Cell Res.

[97] Massard C, Zermati Y, Pauleau A-L, Larochette N, Métivier D, Sabatier L (2006). hTERT: a novel endogenous inhibitor of the mitochondrial cell death pathway. Oncogene.

[98] Jin X, Beck S, Sohn Y-W, Kim J-K, Kim S-H, Yin J (2010). Human telomerase catalytic subunit (hTERT) suppresses p53-mediated anti-apoptotic response via induction of basic fibroblast growth factor. Exp Mol Med.

[99] Ci X, Li B, Ma X, Kong F, Zheng C, Björkholm M (2015). Bortezomib-mediated down-regulation of telomerase and disruption of telomere homeostasis contributes to apoptosis of malignant cells. Oncotarget.

[100] Ning H, Li T, Zhao L, Li T, Li J, Liu J (2006). TRF2 promotes multidrug resistance in gastric cancer cells. Cancer Biol Ther.

[101] Blanco R, Muñoz P, Flores JM, Klatt P, Blasco MA (2007). Telomerase abrogation dramatically accelerates TRF2-induced epithelial carcinogenesis. Genes Dev.

[102] Liu H, Wang H, Li C, Zhang T, Meng X, Zhang Y (2016). Spheres from cervical cancer cells display stemness and cancer drug resistance. Oncol Lett.

